# Chromosome-level genome assembly of the bar-headed goose (*Anser indicus*)

**DOI:** 10.1038/s41597-022-01801-9

**Published:** 2022-11-03

**Authors:** Yawen Zhang, Bo Zhang, Ying Zhang, Ruixue Nie, Jian Zhang, Peng Shang, Hao Zhang

**Affiliations:** 1 National Engineering Laboratory for Animal Breeding, Plateau Animal Genetics Research Center, China Agricultural University, Beijing, 100193 China; 2 Institute of Animal Science, Wenzhou Academy of Agricultural Sciences, Wenzhou, 325006 China; 3 China Agricultural Museum, Beijing, 100125 China; 4 College of Animal Science, Tibet Agriculture and Animal Husbandry College, Linzhi, 860000 China

**Keywords:** Genome, Genomics

## Abstract

Bar-headed geese (*Anser indicus*) are adaptable to plateau environments. In this study, we sequenced and assembled a high-quality chromosome-level genome of the bar-headed goose using PacBio long reads and Hi-C technique, and generated 115.73 Gb of Illumina short-reads and 95.89 Gb of PacBio long-reads. The assembled bar-headed goose genome, with a contig N50 of 5.734 Mb and a scaffold N50 of 65.77 Mb, is 1.129 Gb in length and includes 33 chromosomes and 451 fragments. BUSCO assessment yielded a completeness score of 94.4%. In total, 15,376 protein-coding genes were predicted, of which 94.95% had homologs in protein databases. We identified 78 positively selected genes (PSGs) in the bar-headed goose genome, which were mainly enriched in calcium ion and ATP-binding. This bar-headed goose genome will be an important resource for increasing our understanding regarding the genetic basis of adaptation to life at a high altitude.

## Abstract (summary)

**Table:** 

Measurement(s)	Response to hypoxia
Technology Type(s)	PacBio long reads and Hi-C technique

## Background & Summary

Bar-headed geese (*Anser indicus*) are the highest-flying birds in the world and are common summer migratory birds on the Qinghai-Tibet Plateau. These birds spawn at high altitudes in summer and migrate to lower altitudes in winter^1–3^. Their migration route starts from the winter habitats in India and Nepal, as well as the Yunnan, Guizhou, and Yarlung Zangbo rivers in China to the summer spawning grounds on the Tibetan Plateau of China, Kyrgyzstan, Mongolia, and other countries. They mainly breed and nest on the Tibetan Plateau^4,5^. The difficulty in this migratory flight is to the need to fly over a large natural barrier, “the roof of the world,” and the Qinghai-Tibet Plateau. It is a stunning feat that bar-headed geese (*Anser indicus*) can fly over the Himalayas^6^. Bar-headed geese can reach a height of 5,000–8,000 m during their biannual migration, where the partial pressure of oxygen (PO_2_) is only one-third to half of that at sea level^7^.

How do bar-headed geese fly over the Himalayas? Molecular and physiological mechanisms of adaptation to hypoxia have been previously described in bar-headed geese. The molecular evolution of cytochrome C oxidase promotes mitochondrial energy metabolism; further, the O_2_ transport capacity of bar-headed geese is more adapted to altitude flight^8^. Bar-headed geese are also known to reduce their flight metabolic rates to fly under low oxygen conditions^9,10^. However, the evolutionary mechanism of hypoxic adaptation in this species remains unclear. A high-quality genome is essential for understanding high-altitude adaptation in bar-headed geese. Although a draft scaffold-level genome assembly of bar-headed geese was previously released, it was assembled based on Illumina short-read sequence technology with limited contiguity and quality^11^.

In this study, we applied long-read sequencing (Pacbio), short paired-end reads (Illumina), and Hi-C technology to generate a high-quality chromosome-level assembly of bar-headed goose genome. The final assembled genome was 1.129 Gb in length, containing 1,429 contigs (N50 = 5.734 Mb) and 486 scaffolds (N50 = 65.77 Mb); further, 15,376 protein-coding genes were annotated in the genome. We also explored the evolutionary mechanism of hypoxia adaptation by *de novo* sequencing of the bar-headed geese genome and comparative genome analysis. This genome will provide an essential reference and facilitate understanding of the evolutionary mechanism of hypoxia adaptation in bar-headed geese.

## Methods

### Ethics statement

The sampled geese and experimental procedures in this study were approved by the State Key Laboratory for Agro-Biotechnology of China Agricultural University (Permit Number: XK257).

### Sample collection and genomic DNA sequencing

The blood of a female bar-headed goose was collected from a farm in Shannan, Tibet, China. Genomic DNA was isolated using standard phenol-chloroform extraction. A short fragmented library was prepared with an insert size of 350 bp and sequenced using Illumina HiSeq X Ten to generate 150-bp paired-end reads. Size-selected SMRTbell libraries were prepared with a minimum fragment length cut-off between 10–40 kb. Large insert libraries were sequenced using the PacBio Sequel system. After trimming the low-quality reads and adaptor sequences from the generated raw data, 115.73 Gb of Illumina data and 95.89 Gb of PacBio data were obtained. The N50 of PacBio subreads was 18.51 kb.

### Hi-C library preparation and sequencing

The blood of a bar-headed goose was fixed with formaldehyde and glycine was added to quench the crosslinking reaction. After cell lysis, a four-cutter restriction enzyme (MboI) was used to digest the cross-linked DNA. The DNA ends were then marked with biotin-14-dCTP, and blunt-end ligation of the cross-linked fragments was performed. DNA was isolated using a phenol-chloroform procedure. Fragments were sheared to 100–500 bp sizes by sonication. Fragment ends were repaired using a mixture of T4 DNA polymerase, T4 polynucleotide kinase, and Klenow DNA polymerase. Biotin-labelled Hi-C samples were enriched using streptavidin magnetic beads. A-tailing was added to the fragment ends using Klenow (exo-) and an Illumina paired-end sequencing adapter was added using ligation. Hi-C libraries were amplified using 10–12 cycles of PCR and sequenced on an Illumina HiSeq instrument with 2 × 150 bp reads. After filtering low-quality reads and adaptors with the same standard described above, we obtained 394,408,656 paired-end clean reads for further genome assembly.

### Transcriptome sequencing

Tissue samples from the heart, liver, lung, kidney, brain, and muscle of the female goose were collected for full-length transcriptome sequencing. RNA samples pooled from these tissues were used to construct a library. Using the Clontech SMARTer PCR cDNA Synthesis Kit (Takara Biotechnology, Dalian, China), 3 μg of RNA was transcribed to cDNA and subsequently amplified to generate double-stranded cDNA. cDNA was then size-selected for < 4 kb and > 4 kb fractions using the BluePippin™ Size Selection System (Sage Science, Beverly, MA, USA). Each SMRTbell library was constructed using 1 μg of size-selected cDNA with the Pacific Biosciences SMRTbell Template Prep Kit. The binding of the SMRTbell templates with polymerases was conducted using the Sequel Binding Kit, followed by primer annealing. This generated 21.18 Gb PacBio subreads with an N50 length of 69.4 kb. Sequencing was performed by Annoroad Gene Technology Company on the Pacific Bioscience Sequel platform.

RNA isolated from each tissue was used for mRNA-seq library construction. Poly(A) mRNA isolation, first-strand and second-strand cDNA synthesis, fragment and adapter ligation, and cDNA library preparation were performed sequentially using a TruSeq RNA Sample Prep Kit (Cat. #RS-122-2002; Illumina, San Diego, CA, USA) according to the manufacturer’s instructions. All libraries were sequenced using an Illumina HiSeq platform for PE-150 sequencing. After filtering the low-quality reads and adaptor sequences, we obtained 71.436, 67.420, 68.376, 66.231, 69.447, and 71.265 Mb clean reads from the heart, liver, lung, kidney, brain, and muscle tissues, respectively.

### Genome size estimation

**Fig. 1:**
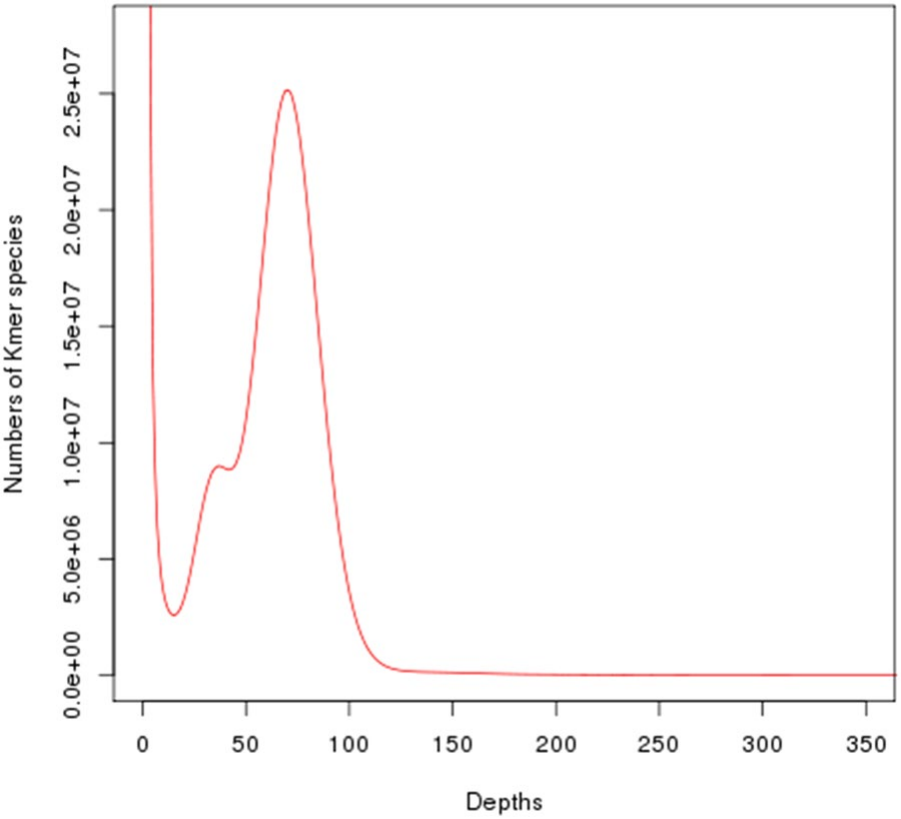
K-mer frequency distribution. Frequency of each K-mer in raw sequencing reads was calculated; here K = 21.

We estimated the genome size of the bar-headed goose using Illumina short reads based on the k-mers method^12^. The total number of k-mers was 88,708,842,375 and the expected k-mer depth was 73 (Fig. 1). Based on a 21-mer analysis, we determined the genome size to be 1142.45 Mb, with a heterozygosity of 0.54% and repeat content of 13.20%.

### Contig assembly and quality assessment

*De novo* assembly of PacBio reads was performed using wtdbg2^13^. Blasr was applied for aligning subreads to the assembled genome sequence with parameters (--bam --bestn 5 --minMatch 18 --nproc 4 --minSubreadLength 1000 --minAlnLength 500 --minPctSimila rity 70 -minPctAccuracy 70 --hitPolicy randombest --randomSeed 1)^14^. Arrow software was used to polish the base-calling of the contigs to remove INDEL errors within the assembly contigs. The contigs were then subjected to a round of Pilon error correction using Illumina reads according to the default parameters^15^. We assembled 1,431 contigs with a total length of 1,135 Mb and a contig N50 size of 5,733 kb. After polishing using PacBio reads and Illumina short reads, the final assembled contigs were 1,136 Mb in length, with a contig N50 size of 5,739 kb and a GC content of 42.36%.

**Fig. 2:**
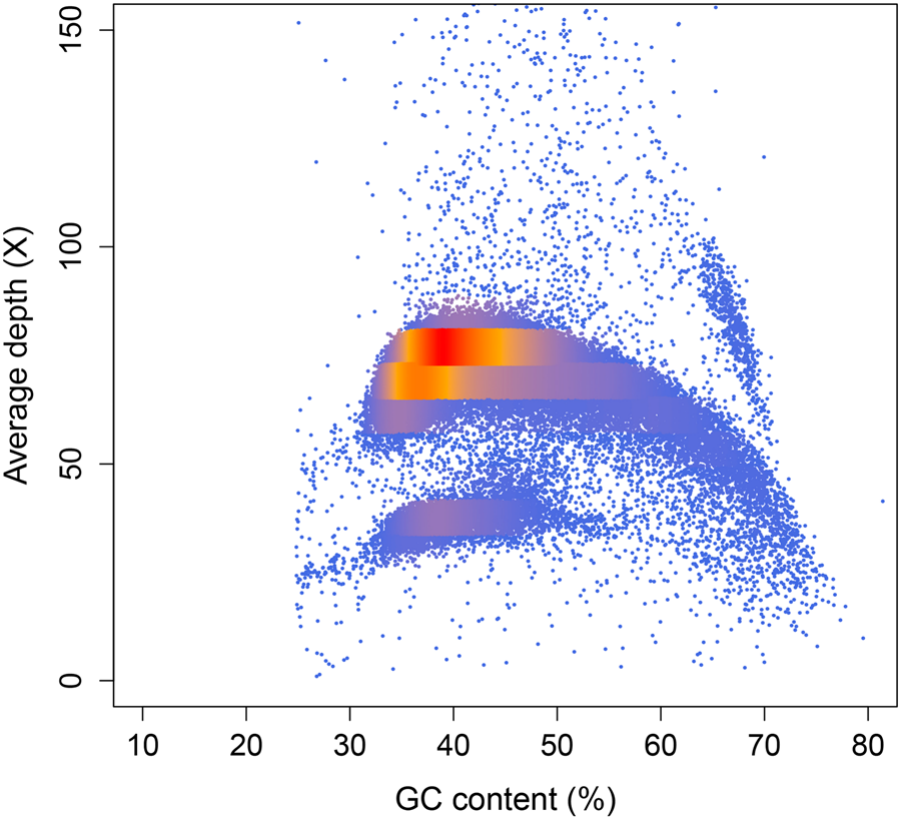
Depth and GC content of the bar-headed goose genome.

After assembly, two methods were used to evaluate the final assembly quality:1) Benchmarking Universal Single-Copy Orthologs (BUSCO v3.0), provides quantitative measures for assessing the genome assembly based on evolutionarily informed expectations of gene content from near-universal single-copy orthologs^16^. The database used was aves_odb9 (4,915 genes). 2) Illumina reads were aligned with the assembled contigs to evaluate completeness based on mapping rates, depth, and coverage. In a total of 4,915 conserved bird BUSCO groups (BUSCO, RRID: SCR 015008), 4,638 (94.4%) were complete (4,581 single-copy(93.2%) and 57 duplicated (1.2%)), 154 (3.1%) were fragmented, and 123 (2.5%) were missing. The contig assembly displayed a well-proportioned distribution of sequencing depth and GC content, indicating that the genome was evenly covered (Fig. 2). Approximately 96.87% of Illumina reads were properly mapped to the contig assembly genome.

### Chromosome anchoring and quality assessment

After filtering the Hi-C data, clean reads were aligned to the reference genome using the bowtie2 end-to-end algorithm^17^. Unmapped reads were mainly composed of chimeric fragments spanning the ligation junction. According to the Hi-C protocol and the fill-in strategy, HiC-Pro (V 2.7.8) could detect the ligation site using an exact matching procedure and to align the 5′ fraction of the reads with the genome^18^. Both mapping steps were then merged into a single alignment file. Low mapping-quality reads, multiple hits, and singletons were discarded. We obtained 266,716,009 unique mapped paired-end reads that were used to construct the scaffolds.

LACHESIS was used to perform clustering, ordering, and orienting^19^. The scaffolds were clustered into N groups using an agglomerative hierarchical clustering algorithm. The longest acyclic spanning tree, called the “trunk,” was built according to the relations between the normalized Hi-C interactions and scaffolds that were excluded from the trunk were reinserted into it at sites that maximized the amount of linkage between adjacent scaffolds. For each chromosome cluster, we obtained the exact scaffold order of the internal groups and traversed all directions of the scaffolds using a weighted directed acyclic graph (WDAG) to predict the orientation of each scaffold. Mummer was used for comparative alignment to identify chromosome Z^20^. The duck was selected as a closely related species in this study. A contact map plotted using HiCPlotter confirmed the genome structure and quality.

**Fig. 3:**
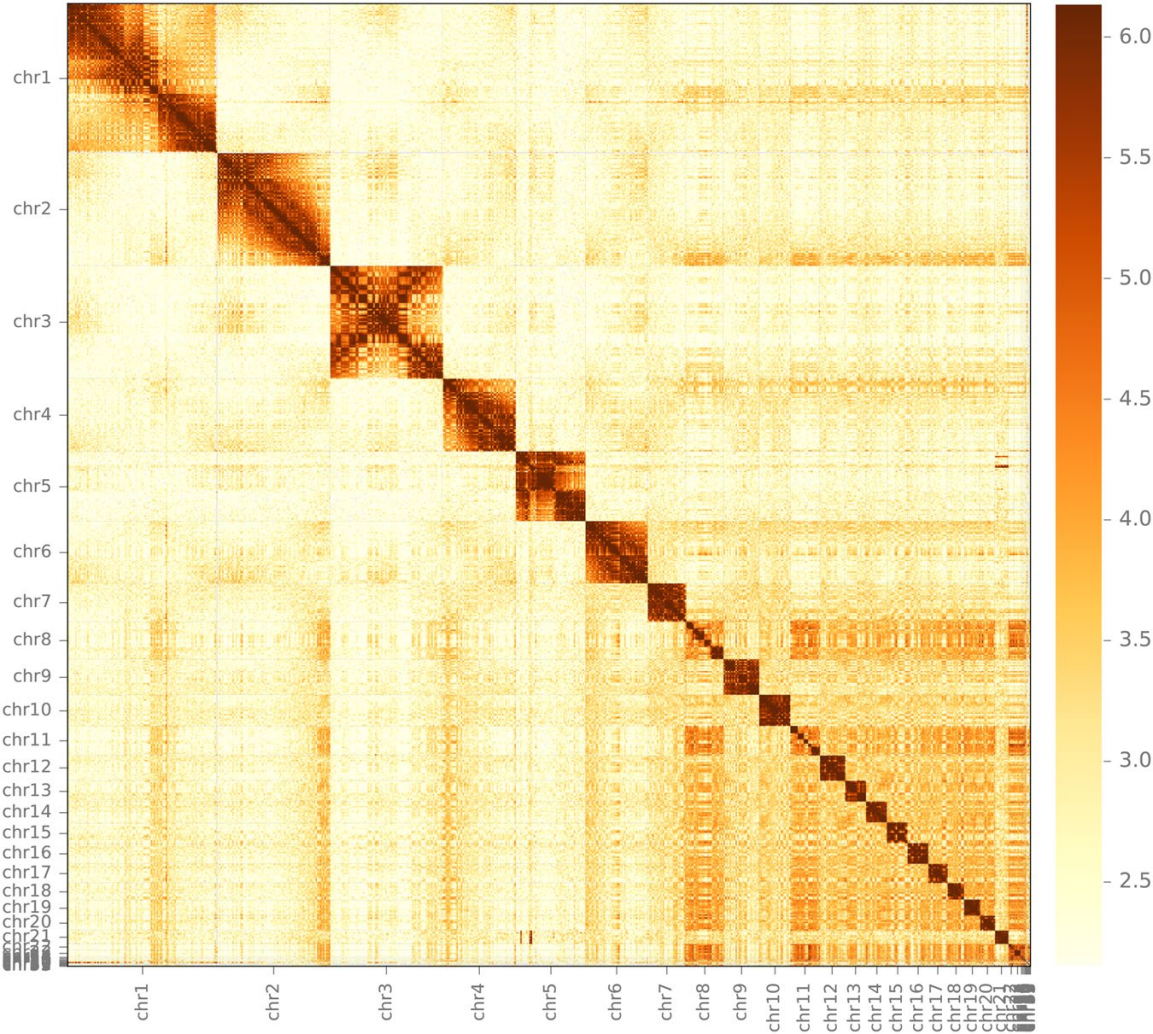
Heatmap of Hi-C interaction density. The scaffolds are split into 500 kb windows, and the interaction density for each pair of windows is measured by the number of supporting Hi-C reads. The interaction density is log-transformed for visualization.

The final assembly contained 486 scaffolds, with a scaffold N50 of 65.77 Mb (Table 1). The scaffolds totalled 1.129 Gb in length, and 1.019 Gb of the scaffold were anchored onto 33 chromosomes, with maximum and minimum lengths of 159.04 Mb and 100.43 kb, respectively (Table 2; Fig. 3). Upon comparative analysis with the duck Z chromosome, the fifth longest chromosome (chr5:74.32 Mb) was determined to be the Z chromosome of the bar-headed goose. The assembled genome of the bar-headed goose was integrated at the chromosomal level as well as with the previous version, which was 1.143 Gb in length with a contig N50 of 120.38 kb and a scaffold N50 of 10.09 Mb^11^.

**Table 1:** Statistics of the bar-head goose genome assembly.

Items	Contig length (bp)	Contig Num	Scaffold length (bp)	Scaffold Num
Total	1,129,361,036	1,429	1,129,455,536	484
N50	5,739,385	56	65,774,817	6
N60	4,087,675	79	39,631,339	8
N70	2,729,061	113	30,691,812	11
N80	1,486,491	168	21,490,641	16
N90	478,571	303	7,022,641	24

**Table 2:** Chromosome sizes and assignment for Hi-C scaffolds.

Pseudomolecule	Contig Num	Length (bp)
chr1	101	159,035,276
chr2	58	120,162,588
chr3	110	119,806,388
chr4	20	78,015,662
chr5 (chr Z)	371	74,329,290
chr6	16	65,774,817
chr7	7	40,986,516
chr8	17	39,631,339
chr9	9	38,243,138
chr10	8	32,774,357
chr11	19	30,691,812
chr12	5	26,882,445
chr13	7	22,590,733
chr14	2	22,262,644
chr15	4	21,964,488
chr16	3	21,490,641
chr17	1	20,169,956
chr18	3	18,072,416
chr19	3	16,404,145
chr20	8	15,461,617
chr21	107	14,290,393
chr22	30	6,279,269
chr23	10	6,571,923
chr24	5	1,306,591
chr25	11	1,196,200
chr26	6	1,114,018
chr27	11	883,039
chr28	5	621,370
chr29	3	890,498
chr30	5	568,456
chr31	3	100,432
chr32	7	658,668
chr33	3	488,247
Total anchored	978	1,019,719,372
Unanchored	451	116,221,017

### Repeats and gene annotations

The repeat sequences in the genome were identified using a combination of *de novo* and homology-based approaches. TRF (v 4.0.6)^21^, RepeatMasker (v. 4.0.6)^22^, and RepeatProteinMask were used to identify and classify different repetitive elements by aligning the *Anser indicus* genome sequences against the Repbase database (RepBase23.12)^23^ using default parameters. We also used RepeatModeler to construct a *de novo* repeat library as the final database, and employed RepeatMasker (v. 4.0.6) to identify and classify repetitive elements in the genome. Finally, after combining the results obtained using the above methods, the total length of the non-redundant repeat sequences after removing the overlapping parts was considered as the total length of the repeat sequences. The results revealed that Repetitive sequences accounted for 12.22% of the genome. Genome-wide search and homology prediction against the Repbase database showed that 11.47% of the bar-headed goose genome belongs to the transposable element (TE) family. The overall repeat content was determined especially for DNA transposons (0.43%), long interspersed nuclear elements (6.23%), small interspersed nuclear elements (0.06%), and long terminal repeats (2.44%).

Gene structures were predicted using three basic strategies: *de novo*, homology-based, and transcriptome sequencing-based prediction. Gene structures supported by the *de novo* prediction software were determined based on the statistical characteristics of genomic sequence data (such as codon frequency and exon-intron distribution). The software used included Augustus (http://augustus.gobics.de/)^24^, SNAP (https://github.com/KorfLab/SNAP)^25^, and GeneMark (http://exon.gatech.edu/GeneMark)^26^. For homology-based gene prediction, the encoded protein sequences of known homologous species (*Apteryx australis, Anser cygnoides, Anas platyrhynchos, Gallus gallus, Homo sapiens, Meleagris gallopavo, and Mus musculus*) were aligned with the genomic sequence of the new species using BLAST (http://blast.ncbi.nlm.nih.gov/Blast.cgi)^27^ and Genewise (https://www.ebi.ac.uk/Tools/psa/genewise)^28^. Evidence supported by transcriptome data, such as EST/cDNA sequences, was used to predict gene structures by genomic alignment using PASA (https://github.com/PASApipeline)^29^. Based on these predictions, we used EvidenceModeler (EVM) (http://evidencemodeler.github.io/)^30^ to integrate the gene sets predicted using various strategies into a non-redundant and complete gene set.

**Fig. 4:**
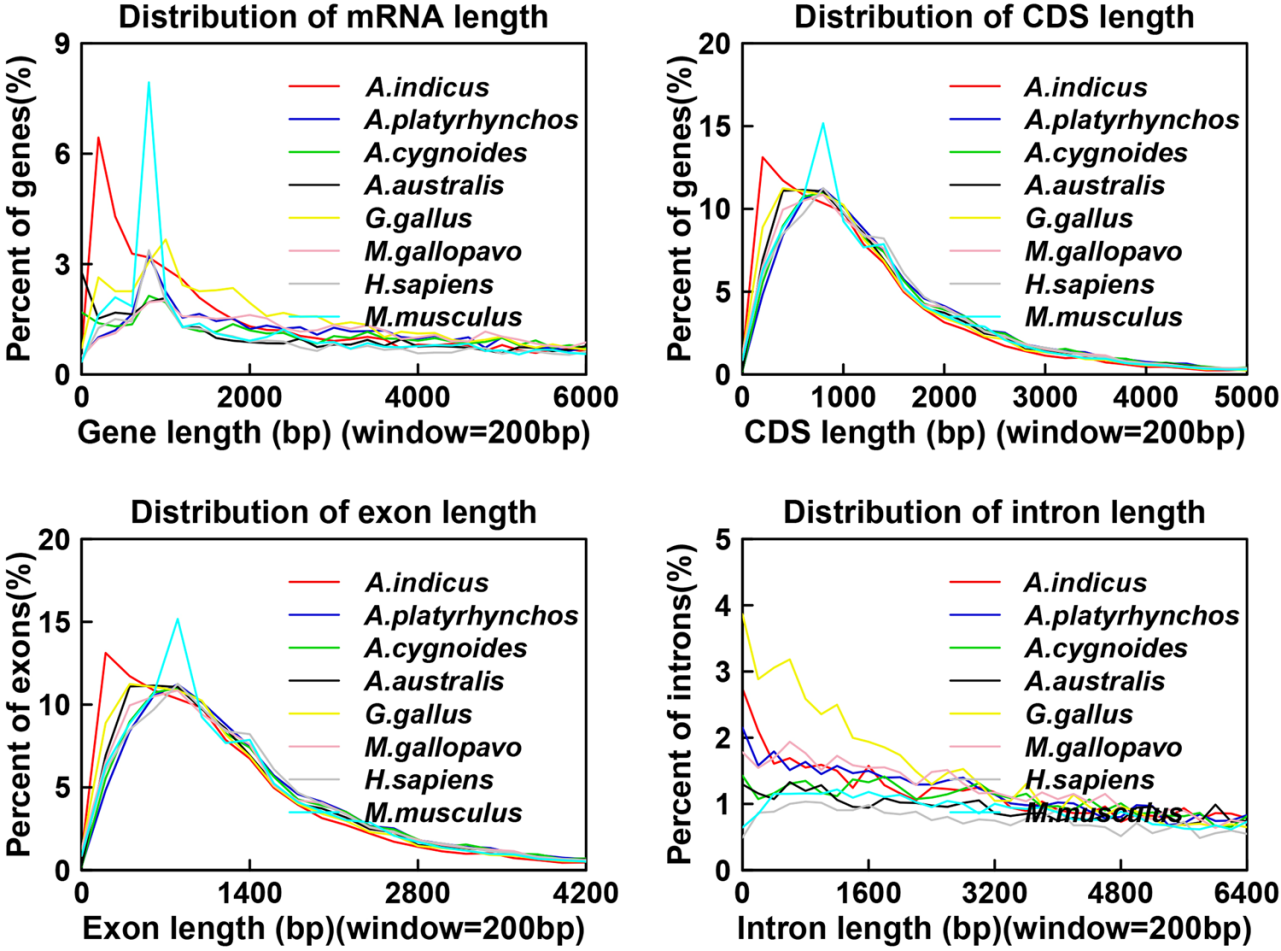
Number of orthologous genes in the bar-headed goose and seven other species.

Through *de novo* prediction using Augustus, Genemark, and SNAP, we identified 24,800, 37,769, and 117,781 protein-coding genes, respectively. The protein-coding genes of homologous species, including *Apteryx australis* (16,687), *Anser cygnoides* (16,574), *Anas platyrhynchos* (16,746), *Gallus gallus* (17,231), *Homo sapiens* (15,518), *Meleagris gallopavo* (15,867), and *Mus musculus* (15,500), were acquired using homology-based prediction. Based on PacBio full-length transcriptomic data, we predicted 73,442 protein-coding genes. We integrated the results of the above three methods to obtain 15,376 protein-coding genes. The average lengths of the genes, exons, and introns were 14,356, 184, and 1,975 bp, respectively. We compared the gene, CDS, exon, and intron lengths with those of seven other homologous species (Fig. 4). Among protein-coding genes, 94.95% had homologs in protein databases including Swissprot (https://web.expasy.org/docs/swiss-prot_guideline.html), NT(https://www.ncbi.nlm.nih.gov/nucleotide/), NR (ftp://ftp.ncbi.nlm.nih.gov/blast/db/FASTA/nr.gz), PFAM (http://xfam.org/)^31^, eggNOG (http://eggnogdb.embl.de/)^32^, GO (http://geneontology.org/page/go-database)^33^, and KEGG (http://www.genome.jp/kegg/)^34^.

Noncoding RNAs (ncRNAs) are a class of RNAs that are not translated into proteins. Four types of ncRNAs were identified in the bar-headed goose genome: microRNAs (miRNAs), transfer RNAs (tRNAs), ribosomal RNA (sRNAs), and small nuclear RNAs (snRNAs). tRNA genes were identified using tRNAscan-SE (v1.3.1)^35^ with default parameters. The rRNA fragments were predicted by aligning the human rRNA sequences with the *Anser indicus* genome sequences using BLASTN with an E value < 1e-5. The miRNA and snRNA genes were searched with BLAST against the Rfam (v13.0) database^36^ using INFERNAL (v1.0)^37^ with the family-specific “gathering” cut-off of Rfam. We annotated 1,611 small ncRNAs, including 564 miRNAs, 253 rRNAs, 483 tRNAs, and 311 snRNAs.

### Gene family identification and specific gene families of the bar-headed goose

This analysis was based on *Anser indicus, Anas platyrhynchos, Anser cygnoides, Gallus gallus, Taeniopygia guttata, Pseudopodoces humilis, Homo sapiens*, and *Mus musculus*. The gene sequences of closely related species were filtered using the following criteria: (1) when there was more than one transcript of a gene, the longest transcript was taken; (2) The protein length was greater than 50 amino acids and all-vs-all BLAST was performed for all protein sequences using the following thresholds: E-value < 10^−10^ and identity >30%. Orthologous gene clusters were classified using hcluster_sq software from OrthoMCL^38^.

**Fig. 5:**
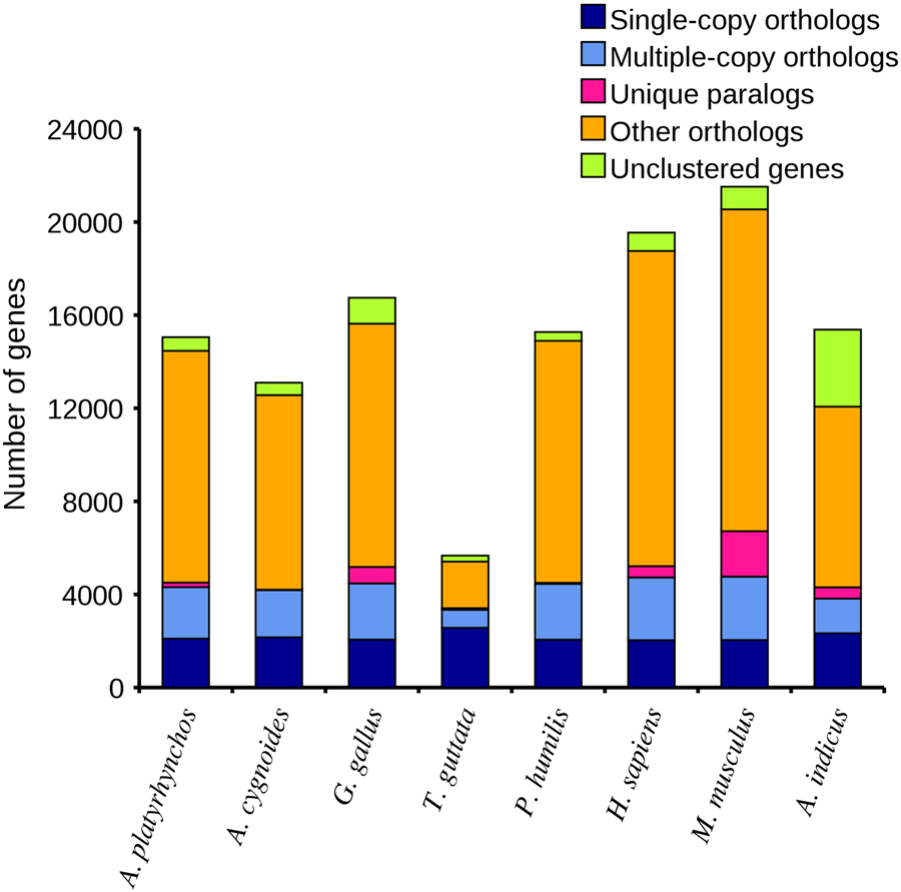
Distribution of genes in different species.

In all, 16,624 gene families were clustered in 8 species. There were 2,904 orthologous gene families shared by all eight species, of which 1,783 were single-copy gene families (Fig. 5). We found that 155 gene families containing 482 genes were specific to the bar-headed goose. Functional annotation of specific genes showed that the top 10 biological process (BP), cellular component (CC), and molecular function (MF) gene ontology (GO) terms were mainly associated with energy metabolism (12 in 30 terms). The specific genes were significantly enriched in 30 KEGG pathways (p ≤ 0.05), which mainly contained ATP-binding cassette transporters, carbon metabolism, and fatty acid metabolism, which are also involved in energy metabolism.

### Phylogenic analysis and divergence time estimation

Single-copy orthologous genes were used to construct a phylogenetic tree. Orthologous protein sequences were aligned using Muscle (v3.6)^39^. Conserved regions were used to construct a maximum likelihood tree with PhyML(v3.0)^40^. The divergence times were estimated based on a set of four-fold degenerate sites from amino acids conserved across all species, using the CDS sequences of single-copy orthologous genes. The MCMCTREE (v 4.5) model of PAML was used to estimate divergence time based on phylogenetic relationships^41^. The fossil calibration times for the divergence were selected based on *Mus musculus-Homo sapiens* (85–97 million years ago [Mya]), *Anas platyrhynchos-Gallus gallus* (75–86 Mya), and *Pseudopodoces humilis-Taeniopygia guttata* (36–46 Mya). MCMCTREE was run to sample 10,000 times, with the sample frequency set to 5,000, after a burn-in of 5,000,000 iterations. The parameters of “finetune” were set as “0.004, 0.016, 0.01, 0.10, and 0.58”. The other parameters were set as the default values.

**Fig. 6:**
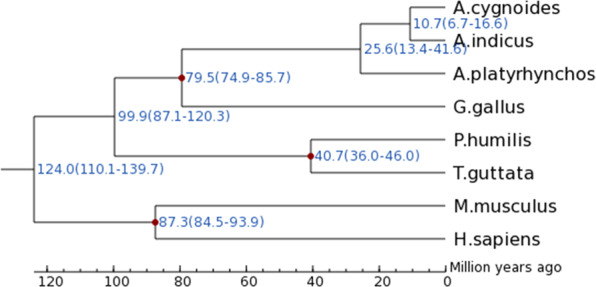
Phylogenetic tree reconstructed using all single-copy orthologs. The scale at the bottom of the figure represents the divergence time. The red dots represent the divergence time and its range (in brackets) between two branches.

From the phylogenetic tree, the swan goose (*A. cygnoides*) was found to be the closest relative of the bar-headed goose. These both belong to the genus *Anser*. The estimated time of divergence between the bar-headed goose and swan goose is approximately 10.7 million years ago (Mya) (Fig. 6). The significant increase in the elevation of the Qinghai-Tibet Plateau occurred approximately 10 million to 8 million years ago. It thus seems reasonable to suggest that the extreme environment of the Tibetan Plateau drove the speciation of bar-headed geese. At the end of the Pliocene or Pleistocene, the species may have begun to migrate from South Asia to Central Asia, although the Himalayas were not yet geologically high. Over time, the migratory route of this bird has been established, and the height of the Himalayas has been increasing. With rising altitudes, bar-headed geese have evolved adaptive mechanisms that allow them to maintain oxygen demand in high-altitude flight.

### Positively selected genes in the bar-headed goose

The dN/dS ratios were calculated for all single-copy orthologs of *Anser indicus* and seven other species. Orthologous genes were aligned using PRANK^42^. Further, ‘codeml’ in the PAML package was employed along with the free-ratio model to estimate the Ka, Ks, and Ka/Ks ratios of different branches. Two models were implemented to test the statistical significance of selective pressure specifically on the ground tit branch: the one-ratio model that acts as the null model (NSsites = 0, model = 0), and model 2 (NSsites = 2). The two models were compared with the LRT calculated from the log likelihood (lnL) values for both models. P-values were obtained by calculating twice the difference between lnLmodel2 and lnLone-ratio and comparing with a chi-square distribution. In total, 78 single-copy orthologous genes in the bar-headed goose revealed a significant positive selection signature. We identified 11 genes (*CASP6, PTHY, VAPB, PK2L2, CHP1, CD36, IBTK, WFS1, LMBD2, KCMB1*, and *MICU1*) that might be involved in GO terms associated with calcium ions, and six of these genes (*NRK2, SUCC, AGK, RENT1, SYL*, and *WSF1*) were annotated as ATP-binding.

### Tissue-specific expression patterns

**Fig. 7:**
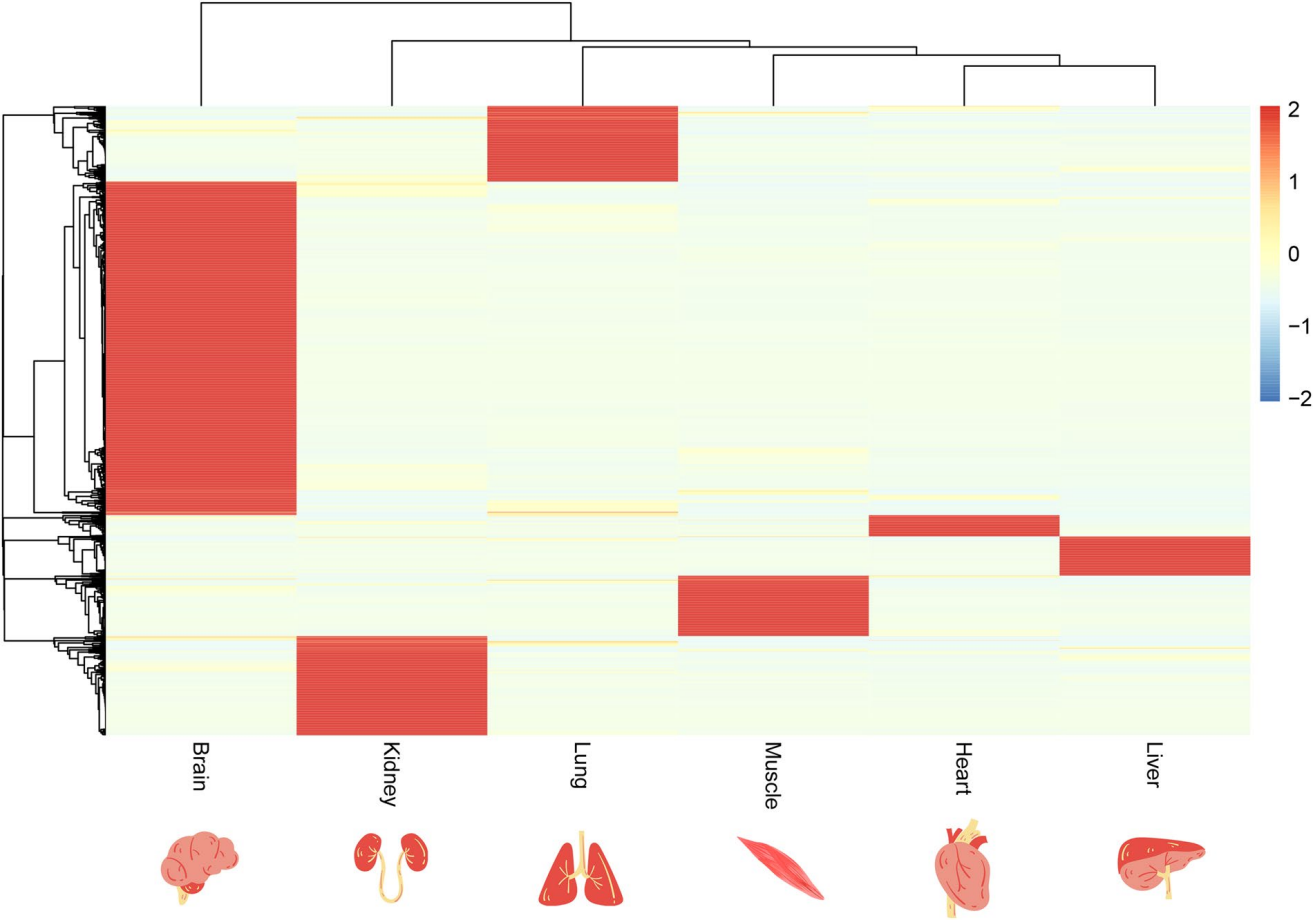
Heatmap of specific-tissue tissue-specific genes.

The RNA-seq transcriptomic data of six tissues (testis, heart, liver, cerebellum, kidney, and brain) were tested for tissue-specific expression patterns in the bar-headed goose. Based on the gene expression values, the Jensen-Shannon divergence (JS score) of genes in tissues was calculated using the information entropy method. The maximum JS score for each gene was considered the tissue-specific score. Genes with a JS score greater than 0.5 were considered tissue-specific expressed genes. We identified 1,591 tissue-specific genes (JS > 0.5) in all six tissues (heart, liver, lung, brain, muscle, and kidney). Gene expression profiles across tissues suggested that the gene expression pattern of the brain is the most different from that of other tissues in this species (Fig. 7). Moreover, most tissue-specific expressed genes (842 genes) were identified in the brain, suggesting that the brain may have different regulatory shifts accompanying the extreme environments.

## Data Records

The raw data were submitted to the Sequence Read Archive (SRA) at National Center for Biotechnology Information (NCBI) database SRP378674^43^. The chromosome-level assembly has been deposited at DDBJ/ENA/GenBank under the accession GCA_025583725.1^44^. The genome annotation results were deposited in the Figshare database^45^.

## Technical Validation

The quality and quantity of total DNA was determined using a NanoDrop 2000 spectrophotometer and a Qubit fluorometer. DNA integrity was determined using an Agilent 2100 Bioanalyzer.

Total RNA was isolated using the TRIzol reagent. RNA integrity was determined using an Agilent 2100 Bioanalyzer (Agilent Technologies, Palo Alto, California, USA). Total RNA samples with a RIN values ≥ 8 were used to construct cDNA libraries for PacBio sequencing.

## Data Availability

All commands and pipelines used for data processing were according to the instruction manuals of the bioinformatics software cited above, and the parameters are clearly described in the Methods section. If no detailed parameters are mentioned for a software, the default parameters were used, as suggested by the developer.

## References

[1] Hawkes LA (2011). The trans-Himalayan flights of bar-headed geese (Anser indicus). Proc Natl Acad Sci USA.

[2] Hawkes LA (2013). The paradox of extreme high-altitude migration in bar-headed geese Anser indicus. Proc Biol Sci..

[3] Bishop CM (2015). The roller coaster flight strategy of bar-headed geese conserves energy during Himalayan migrations. Science.

[4] Zhang J (2020). Assessing site-safeguard effectiveness and habitat preferences of Bar-headed Geese (Anser indicus) at their stopover sites within the Qinghai-Tibet Plateau using GPS/GSM telemetry. Avian Res..

[5] Weigmann C, Lamprecht J (1991). Intraspecific nest parasitism in bar-headed geese, Anser indicus. Anim. Behav..

[6] Swan LW (1970). Goose of the Himalayas. Nat Hist.

[7] Scott GR (2015). How bar-headed geese fly over the Himalayas. Physiol..

[8] Scott GR (2011). Molecular evolution of cytochrome C oxidase underlies high-altitude adaptation in the bar-headed goose. Mol Biol Evol.

[9] Harrison J (2019). The highs and lows of bird flight. Elife.

[10] Meir JU (2019). Reduced metabolism supports hypoxic flight in the high-flying bar-headed goose (Anser indicus). Elife.

[11] Wang W (2020). First de novo whole genome sequencing and assembly of the bar-headed goose. PeerJ.

[12] Liu B (2013). Estimation of genomic characteristics by analyzing k-mer frequency in de novo genome projects. arXiv.

[13] Ruan J, Li H (2020). Fast and accurate long-read assembly with wtdbg2. Nat Methods.

[14] Chaisson MJ, Tesler G (2012). Mapping single molecule sequencing reads using basic local alignment with successive refinement (BLASR): application and theory. BMC Bioinformatics.

[15] Walker BJ (2014). Pilon: an integrated tool for comprehensive microbial variant detection and genome assembly improvement. PLoS One.

[16] Simão FA, Waterhouse RM, Ioannidis P, Kriventseva EV, Zdobnov EM (2015). BUSCO: Assessing genome assembly and annotation completeness with single-copy orthologs. Bioinformatics.

[17] Langmead B, Salzberg SL (2012). Fast gapped-read alignment with Bowtie 2. Nat Methods.

[18] Servant N (2015). HiC-Pro: an optimized and flexible pipeline for Hi-C data processing. Genome Biol.

[19] Burton JN (2013). Chromosome-scale scaffolding of de novo genome assemblies based on chromatin interactions. Nat Biotechnol.

[20] Kurtz S (2004). Versatile and open software for comparing large genomes. Genome Biol.

[21] Benson G (1999). Tandem repeats finder: a program to analyze DNA sequences. Nucleic Acids Res..

[22] Tarailo‐Graovac M, Chen N (2009). Using RepeatMasker to Identify Repetitive Elements in Genomic Sequences. Curr. Protoc. Bioinforma..

[23] Jurka J (2005). Repbase Update, a database of eukaryotic repetitive elements. Cytogenet Genome Res.

[24] Stanke M, Steinkamp R, Waack S, Morgenstern B (2004). AUGUSTUS: a web server for gene finding in eukaryotes. Nucleic Acids Res.

[25] Korf I (2004). Gene finding in novel genomes. BMC Bioinformatics.

[26] Ter-Hovhannisyan V, Lomsadze A, Chernoff YO, Borodovsky M (2008). Gene prediction in novel fungal genomes using an ab initio algorithm with unsupervised training. Genome Res.

[27] Scott M, Madden TL (2004). BLAST: at the core of a powerful and diverse set of sequence analysis tools. Nucleic Acids Res..

[28] Birney E, Clamp M, Durbin R (2004). GeneWise and Genomewise. Genome Res.

[29] Roberts A, Pimentel H, Trapnell C (2011). Identification of novel transcripts in annotated genomes using RNA-Seq. Bioinformatics.

[30] Haas BJ (2008). Automated eukaryotic gene structure annotation using EVidenceModeler and the Program to Assemble Spliced Alignments. Genome Biol..

[31] Finn RD (2014). Pfam: the protein families database. Nucleic Acids Res..

[32] Powell S (2012). eggNOG v3.0: orthologous groups covering 1133 organisms at 41 different taxonomic ranges. Nucleic Acids Res..

[33] Ashburner M (2000). Gene Ontology: tool for the unification of biology. Nat Genet..

[34] Kanehisa M, Goto S, Sato Y, Furumichi M, Tanabe M (2012). KEGG for integration and interpretation of large-scale molecular data sets. Nucleic Acids Res..

[35] Lowe TM, Eddy SR (1997). tRNAscan-SE: A Program for Improved Detection of Transfer RNA Genes in Genomic Sequence. Nucleic Acids Res..

[36] Griffiths-Jones S (2005). Rfam: Annotating non-coding RNAs in complete genomes. Nucleic Acids Res..

[37] Nawrocki EP, Kolbe DL, Eddy SR (2009). Infernal 1.0: Inference of RNA alignments. Bioinformatics.

[38] Li L, Stoeckert CJ, Roos DS (2003). OrthoMCL: Identification of ortholog groups for eukaryotic genomes. Genome Res..

[39] Edgar RC (2004). MUSCLE: Multiple sequence alignment with high accuracy and high throughput. Nucleic Acids Res..

[40] Guindon S (2010). New algorithms and methods to estimate maximum-likelihood phylogenies: Assessing the performance of PhyML 3.0. Syst. Biol..

[41] Yang Z (2007). PAML 4: Phylogenetic analysis by maximum likelihood. Mol. Biol. Evol..

[42] Benavides E, Baum R, McClellan D, Sites JW (2007). Molecular phylogenetics of the lizard genus microlophus (squamatai tropiduridae): Aligning and retrieving indel signal from nuclear introns. Syst. Biol..

[43] (2022). NCBI Sequence Read Archive.

[44] (2022). NCBI Assembly.

[45] Zhang Y (2022). figshare.

